# Predictive value of tumor recurrence using urinary vascular endothelial factor levels in patients receiving radiation therapy for Glioblastoma Multiforme (GBM)

**DOI:** 10.1186/2050-7771-1-29

**Published:** 2013-10-31

**Authors:** Andra V Krauze, Minhee Won, Christian Graves, Ben W Corn, Thierry M Muanza, Steven P Howard, Arul Mahadevan, Christopher J Schultz, Michael L Haas, Minesh P Mehta, Kevin A Camphausen

**Affiliations:** 1 National Cancer Institute/NIH, Bethesda, MD, USA; 2 RTOG Statistical Center, Philadelphia, PA, USA; 3 Tel-Aviv Medical Center, Tel-Aviv, Israel; 4 McGill University, Montreal, QC, Canada; 5 University of Wisconsin Hospital, Madison, WI, USA; 6 Dartmouth Hitchcock Medical Centre, Lebanon, NH, USA; 7 Medical College of Wisconsin, Milwaukee, WI, USA; 8 Reading Health System, West Reading, PA, USA; 9 Northwestern Memorial Hospital, Chicago, IL, USA; 10 Radiation Oncology Branch, National Cancer Institute, NIH, 10 Center Drive, MSC 1682, Building 10, CRC, Rm B2-3500, Bethesda, MD 20892, USA

**Keywords:** VEGF (vascular endothelial factor), Glioblastoma Multiforme (GBM), Urinary biomarker, Radiation therapy, Tumor recurrence

## Abstract

**Background:**

Glioblastoma Multiforme (GBM) is the most common primary malignant tumor of the central nervous system. Standard of care includes maximal resection followed by chemoradiotherapy. Tumors need adequate perfusion and neovascularization to maintain oxygenation and for removal of wastes. Vascular endothelial growth factor (VEGF) is a well characterized pro-angiogenic factor. We hypothesized that the increases in urinary VEGF levels would occur early in the course of tumor recurrence or progression. We examine the feasibility of collecting and analyzing urinary VEGF levels in a prospective, multi-institutional trial (Radiation Therapy Oncology Group, RTOG, 0611) as well as the role of VEGF as a marker of tumor recurrence.

**Method:**

We evaluated VEGF levels in urine specimens collected post-operatively, at the conclusion of radiation therapy (RT) and one month following RT. Urinary VEGF levels were correlated with tumor progression at one year. VEGF levels were measured by enzyme-linked immunosorbant assay in urine specimens and normalized to urinary creatinine levels. Sample size was determined based on a 50% 1-year recurrence rate. With a sensitivity and specificity of 80%, the expected 95% confidence interval was (0.69, 0.91) with 100 patients. A failure was defined as documented disease progression, recurrence or death before one year.

**Results:**

202 patients were enrolled between February-2006 and October-2007. Four patients were ineligible as they did not receive RT. Of the remaining 198 patients, 128 had all three samples collected. In this group, 35 patients (27.3%) did not progress, 89 (69.5%) had progression and 4 (3.1%) died without evidence of progression. Median VEGF levels at baseline were 52.9 pg/mg Cr (range 0.2- 15,034.4); on the last day of RT, 56.6 (range 0–2,377.1); and at one month follow-up, 70.0 (range 0.1-1813.2). In patients without progression at 1-year, both baseline VEGF level and end of RT VEGF level were lower than those of patients who progressed: 40.3 (range 0.2-350.8) vs. 59.7 (range 1.3-15,034.4) and 41.8 (range 0–356.8) vs. 69.7 (range 0–2,377.1), respectively. This did not reach statistical significance. Comparison of the change in VEGF levels between the end of RT and one month following RT, demonstrated no significant difference in the proportions of progressors or non-progressors at 1-year for either the VEGF increased or VEGF decreased groups.

**Conclusion:**

Urine can be collected and analyzed in a prospective, multi-institutional trial. In this study of patients with GBM a change in urinary VEGF levels between the last day of RT and the one month following RT did not predict for tumor progression by one year.

## Background

Glioblastoma Multiforme (GBM) is the most frequent primary malignant brain tumor in adults [1,2]. Its treatment generally includes maximally safe surgical resection followed by adjuvant radiation therapy and temozolomide chemotherapy [3]. Despite advances in understanding the biology behind GBM [1,4,5] and some improvement in outcome with the addition of temozolomide chemotherapy, its prognosis remains grim with a median overall survival (OS) of 15 months [6].

Personalized therapy based on tumor biology in individual patients to improve treatment outcome has identified several potential biomarkers [5,7,8], including vascular endothelial growth factor (VEGF) [9]. Increased VEGF activity has been associated with early recurrence in patients with cancer including GBM [10-12]. VEGF can be measured in biofluids such as urine, serum or plasma [13-15]. The measurement of VEGF in urine is an attractive option as urine collections are noninvasive, there are minimal preparation costs and urinary proteins are stable during long term storage [16,17]. In addition, the measurement of VEGF in serum or plasma can be problematic as VEGF can be released by platelets in the process of phlebotomy in the case of serum [15] and sequestration of VEGF within platelets may make accurate VEGF measurement difficult in the case of plasma [13,14]. VEGF as a urinary biomarker has previously been shown to be a predictive marker for progression free survival (PFS) in cancer patients after the completion of radiotherapy (RT) [10]. Its potential importance to the treatment of GBM is based on its role in pro-angiogenesis.

VEGF is responsible for increased vascularity and vascular permeability in tumors as well as migration and proliferation of endothelial cells [18,19]. VEGF mRNA has been identified in pseudopalisading cells around necrotic zones in GBM and its expression correlates with grade in diffuse astrocytomas [20]. We hypothesized that increased urinary VEGF levels would predict for recurrence or progression in patients with GBM patients. The purpose of this study was to examine the feasibility of collecting and analyzing urine in a prospective, multi-institutional trial and the potential utility of urinary VEGF as a predictive biomarker for progression. In order to explore the dynamic nature of this potential relationship, VEGF was measured in the urine of GBM patients prior to radiation therapy, at completion and at 1 month follow-up.

## Materials and methods

### Specimen and data collection

Under institutional review board approval, following informed consult, voided urine samples were collected from 202 subjects with a diagnosis of primary GBM between February 2006 and October 2007 on RTOG study 0611. This study was approved by the National Cancer Institute Institutional Review Board. A patient was considered eligible if he/she was enrolled on an RTOG GBM study (RTOG 0513 or RTOG 0525). RTOG 0525 is a two arm study in which patients were randomized to receive either standard adjuvant temozolomide therapy or dose dense adjuvant temozolomide [21]. RTOG 0513 is a phase I/II trial in which patients received Motexafin Gadolinium in one of 3 dose levels in phase I, followed by the Motexafin Gadolinium maximum tolerated dose in phase II in conjunction with standard treatment [22]. Among 202 enrolled, 4 patients were subsequently considered ineligible as they did not receive radiation therapy. Of these 198 patients, adequate specimen for analysis at all three time points was available for 128 patients. The urine collection protocol and the need for adequate specimens and analysis have been previously described [16,17]. A minimum of 5 cc of urine was collected from each patient at baseline, at the completion of radiation therapy, and at the 1 month follow-up evaluation.

### Specimen processing and analysis

Urine samples were sent by overnight courier covered with dry ice to a central laboratory for processing and storage. They were measured using a commercial ELISA (enzyme-linked immunosorbant assay) system for VEGF (R&D systems) as per previously published material [16,17]. Urine creatinine levels were measured using the Bayer DCA 2000 following standard protocol [16,17].

#### Statistical analysis

Sample size was calculated to determine estimates of sensitivity and specificity for 1 year recurrence. A 50% 1 year recurrence rate was calculated based on prognostic class average of the RTOG Recursive Partitioning Analysis of Gliomas (RPA) [23,24]. With a sensitivity and specificity of 80% for progression, the expected 95% confidence interval was (0.82, 0.98) based on 100 patients. We calculated that 200 patients were required to ensure that at least 100 patients would have usable specimens based on a previous 50% rate of unusable specimens.

The distribution of VEGF was described with mean, standard deviation, median, minimum and maximum, Q1 (first quartile) and Q3 (third quartile). For the purposes of calculating PFS a patient was considered to have failed treatment if there was documented disease before 1 year or if the patient died without disease before 1 year. Chi-square tests were used to test the distribution difference of pretreatment characteristics of this study and RTOG 0525 patients that were not used in this study, and failure status at 1 year by the change in VEGF from the end of radiation therapy to 1 month post radiation therapy. A Receiver Operating Characteristic (ROC) curve was used to determine the best cut off range for predicting recurrence at 1 year. Logistic regression was used to assess the relationship between patient characteristics and recurrence at 1 year. All testing was done at the overall significance level of 0.05. Statistical Analysis System (SAS, Cary, NC) was used to perform these analyses.

## Results

Of 202 enrolled patients the demographic, clinical and pathologic characteristics of the patient population were compared to a control set of patients enrolled in RTOG 0525 that were not enrolled in the current study (Table 1). Compared to this reference, the patients analyzed in the current study differ significantly in the extent of surgical resection and neurologic functional status [23]. Patients on the current study were more likely to have had gross total resection (p = 0.01, χ2) and better neurological function (p = 0.02, χ2) compared to the RTOG 0525 reference population. The most prevalent RPA class in our cohort was class IV (57.8%). Fifty-three percent of patients were unmethylated at the O^6^-methylaguanine DNA methyltransferase (MGMT) promoter and 25% were methylated.

**Table 1:** Demographic and clinicopathologic characteristics of the patient population as compared to the RTOG 0525 population not used in the analysis

	**RTOG 0611 patients with VEGF (n = 128)**	**RTOG 0525 patients not used in the analysis (n = 997)**
Age		
<50	39 (30.5%)	232 (23.3%)
≥50	89 (69.5%)	765 (76.7%)
	**Chi-square p-value = 0.07**	
Gender		
Female	72 (56.3%)	570 (57.2%)
Male	56 (43.8%)	427 (42.8%)
	**Chi-square p-value = 0.84**	
KPS		
60-80	45 (35.2%)	383 (38.4%)
90-100	83 (64.8%)	614 (61.6%)
	**Chi –square p-value = 0.47**	
Surgery		
Biopsy	3 (2.3%)	47 (4.7%)
Partial resection	44 (34.4%)	459 (46.0%)
Total resection	81 (63.3%)	491 (49.2%)
	**Chi-square p-value = 0.01**	
Neurologic function		
No symptoms	55 (43.0%)	309 (31.0%)
Minor symptoms	48 (37.5%)	468 (46.9%)
Moderate symptoms	24 (18.8%)	215 (21.6%)
Severe symptoms	1 (0.8%)	5 (0.5%)
	**Chi-square p-value = 0.02**	
RPA class		
III	30 (23.4%)	175 (17.6%)
IV	74 (57.8%)	602 (60.4%)
V	24 (18.8%)	220 (22.1%)
	**Chi-square p-value = 0.24**	
MGMT status		
Methylated	31 (25.0%)	269 (27.0%)
Unmethylated	66 (53.2%)	527 (52.9%)
Unknown (indeterminate, invalid)/not done	27 (21.8%)	201 (20.2%)
	**Chi-square p-value = 0.86**	
Treatment arms		
Not randomized	28 (21.9%)	264 (26.5%)
Arm 1	58 (45.3%)	353 (35.4%)
Arm 2	42 (32.8%)	380 (38.1%)
	**Chi-square p-value = 0.09**	

*Q1* first quartile, *Q3* third quartile. *RPA* recursive partitioning analysis.

At 1 year, 35 (27.3%) patients had not progressed, 89 (69.5%) had progressed, and 4 (3.1%) died without progression. For the entire group of 128 patients, at baseline the median VEGF level was 52.9 pg/mgCr (range 0.2-15,034.4); on the last day of RT 56.6 pg/mgCr (range 0 – 2,377.1); and at 1 month following RT 70.0 pg/mgCr (range 0.1-1,813.2). In patients who had not progressed at 1 year, both their baseline VEGF levels of 40.3 pg/mgCr (range 0.2-350.8) as well as their end of RT VEGF levels of 41.8 pg/mgCr (range 0–356.8) were lower than the levels in patients who had progressed at one year, 59.7 pg/mgCr (range 1.3-15,034.4) and 69.7 pg/mgCr (range 0–2,377.1), respectively. However, the differences among these groups did not reach statistical significance.

**Figure 1:**
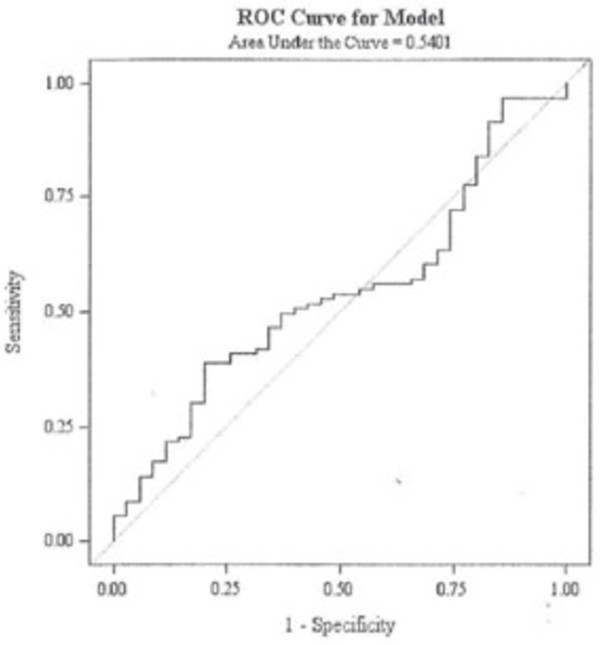
**Receiver Operating Curve (ROC) for urinary VEGF.** Area under receiver Operating curve Curve = 0.5401.

The normalized VEGF change from the end of RT to the 1 month post-RT measurement was compared between patients who progressed by 1-year vs. those that did not progress (Table 2). Chi-square test revealed no significantly higher rate of failure in patients with elevated VEGF at 1 month radiation therapy (p = 0.21). On univariate and multivariate logistic regression analysis no significant correlations were identified for any of the variables examined (randomization arm on RTOG 0525, methylation status, RPA class, baseline normalized VEGF, and VEGF change from end of radiation therapy to 1 month follow-up) (Table 3). The area under the Receiver Operating Characteristic (ROC) curve (AUC) is 0.5401, suggesting that VEGF is not a useful biomarker to predict recurrence at 1 year (Figure 1).

**Table 2:** Normalized VEGF change from the end of RT to 1 month post RT distributed by failure status at 1 year

	**Not failed (neither progression nor death) (n = 35)**	**Failed (progression or death) (n = 93)**
Normalised VEGF		
Increased	22 (62.9%)	47 (50.5%)
Decreased	13(37.1%)	46 (49.5%)

Chi-square test p = 0.21.

**Table 3:** Univariate and multivariate logistic regression analysis of patient characteristics and urinary VEGF for progression/death status at 1 year

	**Univariate**		**Multivariate**	
**Variable**	**p-value**	**OR Ϯ (95% CI)**	**p-value**	**ORϮ (95% CI)**
(**Bolded** value has **unfavorable** outcome)				
Assigned treatment (Arm 2 vs. **Arm1**)	0.76	0.87 (0.36, 2.13)	0.76	0.86 (0.32, 2.28)
Methylation status (**unmethylated** vs. methylated)	0.25	1.72 (0.68, 4.34)	0.18	2.04 (0.73, 5.75)
RPA (**IV** vs. III)	0.62	1.26 (0.51, 3.14)	0.99	0.99 (0.31,3.12)
RPA (**V** vs. III)	0.17	2.50 (0.67, 9.31)	0.73	1.30 (0.29, 5.90)
Baseline Normalized VEGF (continuous)	0.34	1.001(0.998,1.004)	0.45	1.001 (0.998,1.005)
Normalized VEGF change from End of RT to 1 month post RT (continuous)	0.50	1.000 (0.999,1.001)	0.31	0.999 (0.998,1.001)

Ϯ Odds ratio: the OR of 1 indicates no difference, and > 1 indicates higher failure rate with increasing values.

## Discussion

VEGF has emerged as a significant drug target due to its importance as a pro-angiogenic cytokine in gliomas [4]. VEGF signaling is thought to occur in response to hypoxia and results in the formation of vessels whether *de novo* or from preexisting vasculature [25]. Its role may be especially important in gliomas as they exhibit aberrant vessels, hypoxia and endothelial proliferation. Increasing VEGF activity correlates with an increase in vascular proliferation and increasing tumor grade in gliomas [26]. A number of drugs aimed at neutralizing VEGF receptors are currently being investigated [9], most notably bevacizumab, a monoclonal antibody that binds and neutralizes VEGF. Bevacizumab has been approved for use in non-small cell lung cancer, metastatic renal cell carcinoma, and based on the BRAIN [27] and the NCI 06-C-0064E studies [28] it has also been approved for use in patients with recurrent GBM. The two GBM trials showed a reduction in steroid use, temporary improvement in neurological function and, in the BRAIN study, a PFS of 9.2 months. In the newly diagnosed GBM setting, a phase II trial [29] has shown a PFS benefit with no OS benefit when Avastin is used in conjunction with RT and temozolomide, although subset analysis showed that RPA class IV/V may have an OS benefit as well. The addition of Avastin to the standard regimen of radiation and temozolomide is currently being investigated in large randomized phase III trials (RTOG 0825, BO21990).

Previously, Chan et al. [10] showed that urinary VEGF levels at the end of radiotherapy compared to the level at least 1 month post treatment to be predictive of 1 year PFS in a variety of tumor types, including GBM. Thus, the present study focused on VEGF as a potential biomarker for tumor recurrence in patients with GBM receiving chemoradiation. Our results show that the patient population in the current study is largely representative of the RTOG0525 population, suggesting applicability to GBM patients in general. However, we measured no significant difference with respect to changes in VEGF in the patients who failed treatment and those who did not. Furthermore, no significant correlation was found on univariate/multivariate analysis for either baseline VEGF or the change in VEGF level. However only 25% of patients were found to have methylated MGMT in the current study as compared to historical rates on the order of 45% [5] to 50% [30-32]. As methylated MGMT has been shown to be both prognostic and predictive for an improved outcome in GBM [33-36], this may affect the survival outcome as patients may be more likely to fail treatment. Recent analysis of the RTOG0525 results does not suggest that this is the case as their outcomes are comparable to published literature [31]. Randomization to either arm was not statistically different suggesting that standard chemotherapy vs. dose dense chemotherapy is likely not a confounding factor with respect to urinary VEGF (Table 1).

Limitations of the study include the small number of patients (128) for whom adequate samples were available as well as potentially the timing of VEGF measurement and the lack of long term follow-up of urinary VEGF levels. Although previous data [10] showed a correlation between urinary VEGF levels and PFS in a population of cancer patients, there is the possibility that the correlation with respect to GBM may exist but is simply not being detected in the current study as result of the timing of VEGF measurement. VEGF secretion in quantities large enough to be predictive for recurrence may not occur until a significant burden of disease is present, which is less likely at the end of treatment or even at the 1 month follow-up point. The possibility remains that VEGF levels could in fact be predictive at a later date that is still before a definitive radiographic diagnosis could be made. In such a scenario, they could potentially help distinguish progression from pseudoprogression.

Despite this lack of statistical significance, baseline values for VEGF were higher for patients who progressed by 1 year. This was the case both at baseline as well as at the end of radiotherapy. This may support the idea that at the very least urinary VEGF may have value as a prognostic if not necessarily as a predictive biomarker in GBM.

Recent studies have revealed significant heterogeneity within gliomas and even within the narrowly defined GBM histology [1,37,38]. Based on the GBM subtypes identified in Verhaak et al., it is possible that VEGF levels may be predictive in some GBM subtypes specifically the mesenchymal or classical. EGFR activation (classical subtype) and PTEN mutation (mesenchymal subtype) have both been associated with increased VEGF expression [4]. Further studies could examine a possible correlation between VEGF levels and molecular GBM subtypes and possibly include VEGF level within the risk classification itself. A possible correlation between GBM subtypes and VEGF was not addressed in the current study. RTOG 0825, one of the two ongoing phase III trials involving bevacizumab, includes patient stratification by mesenchymal/angiogenic gene expression and may indirectly explore this potential relationship.

## Conclusion

Urinary VEGF was not identified as a predictive factor for tumor recurrence in GBM patients receiving radiation therapy. There is a suggestion that baseline and post treatment VEGF levels may be higher in patients who ultimately fail treatment although this was not statistically significant. This study confirmed that urine can be collected and analyzed in a prospective, multi-institutional trial.

## Consent

Written informed consent was obtained from the patients for the publication of this report.

## Competing interests

Dr. Mehta has served as a consultant for Merck, Roche, BMS, Novocure, Elekta, Phillips, Novelos and has stock optins in Accuray and Pharmacyclics.

## Authors’ contributions

All authors contributed equally to work and read and approved the manuscript.
